# Correlation Between Oncostatin M and Acute Ischemic Stroke: A Case-Control Study

**DOI:** 10.7759/cureus.50297

**Published:** 2023-12-10

**Authors:** Michael Christian, Bo Long, Zhanglin Tian, Yuhan Dong, Junmeng Huang, Youdong Wei

**Affiliations:** 1 Department of Neurology, The First Affiliated Hospital of Chongqing Medical University, Chongqing, CHN

**Keywords:** serum, oncostatin m, expression, correlation, biomarker, acute ischemic stroke

## Abstract

Background: The expression of oncostatin M (OSM) has been studied in various diseases related to inflammatory response, but its implementation in acute ischemic stroke (AIS) remains to be explored.

Objective: The objective of this study is to assess the correlation between serum OSM expression and various aspects of AIS in a clinical setting.

Materials and method: A single-centered case-control study was performed in the First Affiliate Hospital of Chongqing Medical University from October 2020 to March 2021. A total of 134 patients were enrolled in the AIS group and 34 healthy individuals were enrolled in the control group. Physical examinations were performed and venous blood samples were collected. Enzyme-linked immunosorbent assay (ELISA) was used to measure serum OSM. Trial of Org 10172 in Acute Stroke Treatment (TOAST) classification, National Institutes of Health Stroke Scale (NIHSS) score, magnetic resonance imaging (MRI) scan, and modified Rankin scale (mRS) were used to assess the classification, etiology, severity, and prognosis of the AIS group. Assessments were done to analyze serum OSM expression based on sensitivity, etiology, severity, prognosis, and several risk factors of AIS. Regression models, correlation, and sensitivity tests were performed to explore the correlation of OSM expression with various aspects of AIS.

Results: There was a statistically significant elevation of serum OSM expression in the AIS group (P<0.001). All AIS subgroups showed elevation in OSM level and statistically significant results were reflected in three subgroups. The area under the curve to differentiate AIS patients and control by serum OSM level was 0.747 (P<0.001), with the optimal cut-off value showing sensitivity at 58.82% and specificity at 75.37%. The elevation of serum OSM expression was proportional with severity, not proportional to the volume of infarct, and less elevated in the favorable outcome group. Serum OSM correlation with several risk factors of AIS was statistically significant in age, low-density lipoprotein, non-high-density lipoprotein, prothrombin time, and systolic blood pressure.

Conclusion: Serum OSM was expressed differently in correlation with various aspects of AIS. Our findings supported the initial hypothesis that OSM is correlated with various aspects of AIS in humans.

## Introduction

Stroke is one of the leading causes of death and disability worldwide, ranked first in China in 2017 [1,2]. Epidemiological studies highlight the global burden of stroke, which greatly affects low- and mid-income countries [3]. Ischemic stroke constitutes 70% of stroke cases with a high chance of long-term recurrence. In 2019, 3.29 million stroke-related mortality were reported, among which 50.3% were caused by ischemic stroke [4]. Acute ischemic stroke (AIS) progresses rapidly in a short time and constitutes 80% of cases in China. China Stroke Surveillance underlined that the increase in the prevalence of stroke in China is due to the aging population in combination with the risk factors of stroke that are poorly controlled [5].

Present identification and management of AIS rely heavily on large instruments and critical care specialists which includes multiple disciplinary approaches [6,7]. This explains the greatest burden of stroke in low- and mid-income countries, as most often time-critical management for stroke is not available due to socioeconomic conditions [8-12]. An integrative approach aimed at developing a widely available and cost-effective alternative could significantly enhance the management of AIS; this has led to a growing interest in exploring potential AIS biomarkers alongside existing methods [7].

Oncostatin M (OSM) is a family of Interleukin-6 (IL-6), a wide-purpose glycoprotein, known due to its ability to participate in the systemic inflammatory response, as well as a broad and often context-dependent action on various cellular processes, ranging from differentiation, hematopoiesis, proliferation, and survival [13]. Studies have reported OSM as a potential biomarker in various types of cancer [14-16] and inflammatory-related diseases [17-19]. Evidence from animal models confirmed the involvement of OSM in ischemic stroke [20], cardiac disease [21], and lipolysis of adipose tissue [22]. Despite the rising evidence of OSM involvement in inflammatory-related disease, its effect on the nervous system is still poorly understood [23]. A study on the long-term proinflammatory effect on AIS reported an elevation of OSM, but the correlation between OSM and the risk factors, severity, and prognosis remains to be explored [24].

Our study aimed to bridge the gap in our current understanding of the correlation between OSM and AIS. We hypothesized that OSM expression is associated with various factors related to AIS in humans. Our goal was to validate previous findings and contribute to the understanding of OSM as a potential biomarker for AIS.

## Materials and methods

Study design and population

This was a single-centered case-control study conducted in the First Affiliated Hospital of Chongqing Medical University, Chongqing, China. The study was approved by the Ethics Committee of the First Affiliated Hospital of Chongqing Medical University (approval number: 2020-593). All participants signed informed consent. A total of 134 patients were included in the AIS group from among patients with AIS admitted to the Department of Neurology of the First Affiliated Hospital of Chongqing Medical University between October 2020 and March 2021. We set the inclusion criteria for AIS as follows: (i) Diagnosis of AIS according to the Chinese Guideline for the Diagnosis and Treatment of AIS 2018 and (ii) onset within 72 hours. The exclusion criteria were: (i) intracranial hemorrhage, (ii) transient ischemic attack, (iii) history of stroke, (iv) degenerative disease of the nervous system, (v) brain tumors, (vi) severe liver and kidney function damage, (vii) history of systemic inflammatory disease, and (viii) lack of brain MRI. A total of 34 individuals were assigned to the control group, derived from healthy individuals who came for physical examination in the same time period. The proportion of sex and age for the control group was matched to the AIS group. People with any acute and/or chronic diagnosis after physical examination were excluded from control group inclusion.

Serum OSM collection and measurement

Both the AIS group and control group went through a physical examination to record the basis of two sample characteristics; 5 ml of cubital venous blood was drawn at the time of admission to measure OSM serum expression and other related characteristics. The collected blood sample was centrifuged at 3000 rpm for five minutes and then the serum was stored at -80°C preceding measurement. Enzyme-linked Immunosorbent Assay (ELISA) kit (Shanghai Kanglang Biotechnology Co., Ltd, Shanghai, China) was used to quantitatively measure OSM in serum in pg/ml.

Clinical data collection and management

The following clinical data were collected from both groups: sex, age, body mass index (BMI), history of hypertension, diabetes, hyperlipidemia, smoking, and alcohol consumption. From a blood sample, the following data were also collected and measured in addition to OSM: fasting blood glucose (FBG), total cholesterol (TC), triglyceride (TG), low-density lipoprotein (LDL), high-density lipoprotein (HDL), non-high-density lipoprotein (non-HDL), homocysteine (HCY), white blood cell (WBC), fibrinogen (Fib), d-dimer (D-D), prothrombin time (PT), and activated partial thromboplastin time (APTT).

Based on Trial of Org 10 172 in Acute Stroke Treatment (TOAST) classifications, the AIS group was differentiated into five groups: (i) large artery atherosclerosis (LAA), (ii) cardioembolic (CE), (iii) small artery occlusion (SAO), (iv) stroke of other determined cause (SOC), and (v) stroke of undetermined cause (SUC). To evaluate the severity of the neurological impairment of the admitted patients, at the time of admission patients were scored per the National Institutes of Health Stroke Scale (NIHSS). The total score was 42: those under 5 points were classified as mild, between 5-15 as moderate, and above 15 were classified as severe. The volume of cerebral infarction was collected by MRI scan in cm^3^. Patients with less than 5 cm^3^ of infarction were classified into the small infarction group, between 5-15 belonged to the medium infarction group, and greater than 15 cm^3^ belonged to the large infarction group. The modified Rankin scale (mRS) was used to assess the functional prognosis of the AIS group at 90 days after onset. The mRS used 0-6 points to describe seven different outcomes from asymptomatic to mortality: 0-2 points were classified as good prognosis, 3-6 points were classified as poor prognosis, and 7 as mortality. The process was done through outpatient follow-up or telephone follow-up.

The entire assessment of TOAST classification, NIHSS score, infarct volume, and mRS was done independently by two neurologists. Under disputable conditions, the final result was obtained after a discussion between the two neurologists.

Statistical methodology

Regression models were used to analyze the influence of variables. The number of categorical variables was shown as case occurrence percentage, n (%). The Shapiro-Wilk Test was used to distinguish whether continuous variables were normal distribution. Continuous variable with normal distributions was represented by mean ± standard deviation (x ®±s), and continuous variables with non-normal distribution were represented by the median and interquartile range, M (Q1, Q3). The χ2 was used to determine whether there was a statistical difference between two groups of categorical variables. The Mann-Whitney U test was used to compare the differences between two groups of non-normal distribution indicators in continuous variables. The t test was used to compare the differences in normal distribution indicators between two groups, and the Kruskal-Wallis H-test was used to compare the differences in non-normal distribution indicators of multiple groups. For variables that are not normally distributed, Spearman Rank Correlation was used to determine the correlation between two variables. The receiver operating characteristics (ROC) curve was used to evaluate the ability of OSM to distinguish between the AIS and control groups. IBM SPSS Statistics for Windows, Version 25.0 (Released 2017; IBM Corp., Armonk, New York, United States) and GraphPad Prism 9 version 9.5.1 (Dotmatics, Boston, Massachusetts, United States) were used to compute the statistics and build figures.

## Results

Baseline comparison of clinical data between AIS and control group

On comparing the two groups, the difference in the sex and age category was found to be not significant (P>0.05). Statistically significant (P<0.05) differences were found for the following categories: hypertension, diabetes, smoking history, alcohol drinking history, FBG, WBC, and D-D (Table 1).

**Table 1:** Baseline and Clinical Data AIS: acute ischemic stroke; APTT: activated partial thromboplastin time; BMI: body mass index; D-D: d-dimer; FBG: fasting blood glucose; HCY: homocysteine; HDL: high-density lipoprotein; LDL: low-density lipoprotein; NIHHS: National Institutes of Health Stroke Scale; PT: prothrombin time; TC: total cholesterol; TG: triglyceride; WBC: white blood cell. n (%) applies to sex, hypertension, diabetes, hyperlipidemia, smoking history, and alcohol drinking history; 𝒙̅ ± 𝒔 applies to BMI and TC; M (Q1, Q3) applies to age, FBG, TG, LDL, HDL, HCY, WBC, fibrinogen, D-D, APTT, NIHSS score, and infarction volume. ^a^ represents the value of χ^2^, ^b^ represents the value of Z, ^c^ represents the value of T, and - indicates none of this.

	AIS group (*n*=134)	Control group (*n*=34)	Test statistics	P-value
Sex, male	77 (57.46%)	20 (58.82%)	0.021^a^	0.886
Age, year	66.50 (57.00, 75.00)	63.50 (52.75, 76.00)	-1.096^b^	0.279
BMI, kg/m^2^	24.54 ± 3.69	23.53 ± 2.61	-1.495^c^	0.137
Hypertension	104 (77.61%)	9 (26.47%)	32.210^a^	0.000
Diabetes	48 (35.82%)	5 (14.71%)	5.599^a^	0.018
Hyperlipidemia	71 (52.99%)	18 (52.94%)	3.888^a^	0.143
Smoking history	58 (43.28%)	9 (26.47%)	6.740^a^	0.034
Alcohol drinking history	31 (23.13%)	3 (8.82%)	7.128^a^	0.028
FBG, mmol/L	7.32 (5.20, 8.80)	5.66 (4.80, 5.80)	-3.076^b^	0.002
TC, mmol/L	4.43 ± 1.04	4.58 ± 1.15	0.724^c^	0,470
TG, mmol/L	1.23 (0.96, 1.79)	1.29 (0.93, 1.93)	-0.310^b^	0.757
LDL, mmol/L	2.75 (2.07, 3.33)	2.82 (2.10, 3.54)	0.527^b^	0.599
HDL, mmol/L	1.18 (0.96, 1.31)	1.08 (0.90, 1.31)	-0.798^b^	0.426
HCY, μmol/L	12.96 (10.13, 15.83)	12.03 (9.40, 14.95)	-1.115^b^	0.268
WBC, ×10^9^/L	7.97 (6.56, 10.38)	6.20 (5.15, 6.68)	-4.517^b^	0.000
Fibrinogen, mg/L	2.84 (2.35, 3.40)	2.97 (2.42, 3.35)	-0.110^b^	0.913
D-D, mg/L	0.58 (0.28, 1.30)	0.26 (0.15, 0.51)	-2.147^b^	0.033
PT, s	11.50 (10.90, 12.30)	11.75 (11.13, 12.40)	-0.278^b^	0.781
APTT, s	26.30 (24.75, 27.95)	27.25 (25.95, 28.45)	1.232^b^	0.220
NIHSS score	4.00 (2.00, 9.00)	-	-	-
Infarction volume, m^3^	1.50 (0.21-13.46)	-	-	-

Comparison of serum OSM in the AIS group and control group

**Figure 1:**
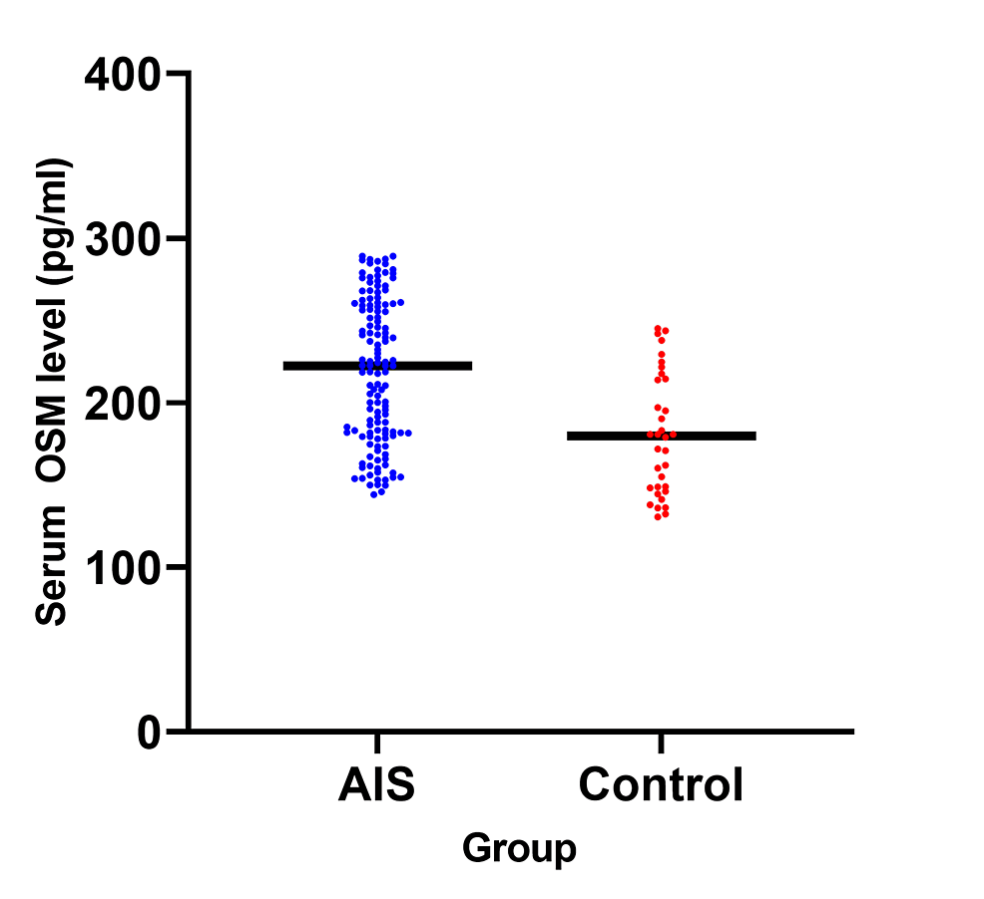
Comparison of serum OSM level between the AIS group and the control AIS: acute ischemic stroke; OSM: oncostatin M

OSM level expression was found to be elevated in the AIS group compared to the control group (AIS= 222.55 (181.38, 259.50) pg/ml vs control= 172.00 (146.10, 217.70) pg/ml), and the result was statistically significant (P<0.001) (Figure 1).

The effectiveness of serum OSM expression for differentiating the AIS group and the control group

**Figure 2:**
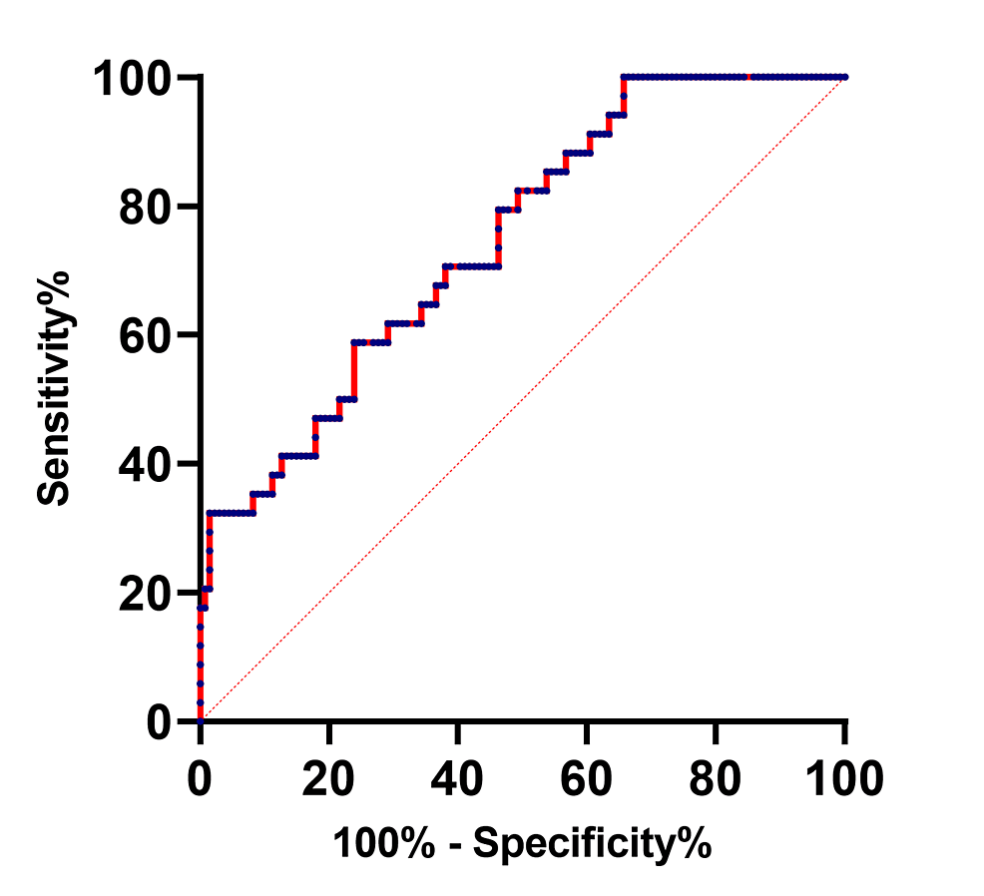
The ROC curve of serum OSM level differentiates between the AIS group and the control group AIS: acute ischemic stroke; OSM: oncostatin M; ROC: receiver operating characteristics

The effectiveness of serum OSM expression in the identification of AIS was evaluated based on ROC curves. The area under the curve (AUC) was 0.7471 (95%CI 0.6598-0.8345, P<0.001} and the optimal cut-off value was 181.5 pg/ml, where the sensitivity was at 58.82% and specificity at 75.37% (Figure 2).

Correlation of serum OSM expression and the etiology of the AIS 

Table 2 displays the five subgroup classifications derived from TOAST. The most significant elevation was expressed in the CE subgroup, followed by SOC, LAA, SAO, and SUC. A statistically significant result was observed in the LAA, CE, and SAO subgroups (P<0.005). Kruskal-Wallis H-test was conducted to find the difference in serum OSM concentration of these five subtypes, and the difference was not statistically significant (P>0.05).

**Table 2:** Etiological subgroup analysis of serum OSM concentration in the AIS group CE: cardioembolic; LAA: large artery atherosclerosis; SAO: small artery occlusion; SOC: stroke of determined cause; SUC: stroke of undetermined cause; OSM: oncostatin M n (%) applies to the amount of each subgroup and M (Q1, Q3) applies to the respective serum OSM level.

	Amount (%)	Serum OSM level (pg/ml)	*Z*-value	P-value
LAA	62 (46.27%)	218.86 (180.85, 289.20)	3.744	<0.001
CE	25 (18.66%)	255.40 (186.70, 278.30)	4.555	<0.001
SAO	39 (29.10%))	210.70 (179.40, 260.20)	3.287	0.005
SOC	4 (2.99%)	222.73 (193.98, 251.55)	1.794	0.364
SUC	4 (2.99%)	190.08 (157.90, 229.83)	0.462	>0.999
*H*-value		6.048		
*P*-value		0.196		

Correlation between serum OSM expression and the severity of neurological deficit and volume of cerebral infarction

We tested the serum OSM expression concerning the degree of severity according to the NIHSS score (Table 3). It was found that serum OSM level expression was higher with the degree of severity of AIS. The severe group had the highest serum OSM expression compared to the mild and moderate groups, where the H-test indicated that the difference in OSM concentration across the three groups was not statistically significant (P>0.05). We further examined the expression correlation with the volume of infarction in the AIS group (Table 4). Among the three categories, we found that the large infarction group had the largest variance, whereas the small and medium infarction had a more similar statistical profile. The H-test revealed that the difference between the three groups was not statistically significant (P>0.05).

**Table 3:** Correlation between serum OSM concentration and severity of neurological deficit in the AIS group NIHSS: National Institutes of Health Stroke Scale; OSM: oncostatin M n (%) applies to the amount of each subgroup and M (Q1, Q3) applies to the respective NIHSS score and serum OSM level.

	Amount (%)	NIHSS score	Serum OSM pg/ml	H-value	P-value
Mild group	74 (55.2%)	2.00 (1.00, 3.00)	215.88 (181.70, 259.75)	0.756	0.685
Moderate group	37 (27.6%)	8.00 (5.00, 11.00)	220.62 (169.75, 260.00)		
Severe group	23 (17.2%)	18.00 (17.00, 21.00)	225.64 (198.00, 259.00)		

**Table 4:** Correlation between serum OSM level and cerebral infarction volume in the AIS group n (%) applies to each infarction category and M (Q1, Q3) applies to the respective infarction volume and serum OSM level. AIS: acute ischemic stroke; OSM: oncostatin M

	Amount (%)	Infarction volume cm^3^	Serum OSM pg/ml	* H*-value	P-value
Small infarction	89 (66.42%)	0.35 (0.043, 1.50)	222.50 (182.40, 259.40)	0.221	0.895
Medium infarction	14 (10.45%)	9.95 (7.50, 12.89)	216.45 (181.05, 257.20)		
Large infarction	31 (23.13%)	76.38 (32.22, 180.43)	226.10 (173.70, 260.50)		

Correlation between serum OSM expression and the prognosis of AIS

We examined the expression of serum OSM level in relationship with patients’ prognostic outcome in the AIS group according to the 90-day mRS score (Table 5). There was no patient assigned to the mortality outcome. We found that the result in the unfavorable outcome group expressed a higher serum OSM level compared to the favorable outcome group. After further examination, the difference in serum OSM level between the two groups was not statistically significant (P>0.05). We used simple logistic analysis to analyze the interdependence, and the result showed that serum OSM was independent of prognosis (OR=0.997, 95%CI 0.9892-1.0051, P=0.488}.

**Table 5:** Correlation between serum OSM expression and the AIS prognosis n (%) applies to the amount of each outcome and M (Q1, Q3) applies to the respective 90-day mRS score and serum OSM level. AIS: acute ischemic stroke; OSM: oncostatin M; mRS: modified Rankin scale

	Amount (%)	90-day mRS score	Serum OSM pg/ml	Z-score	P-value
Favorable outcome	79 (58.96%)	1.00 (0.00, 2.00)	216.70 (181.70, 258.30)	0.083	0.492
Unfavorable outcome	55 (41,04%)	4.00 (3.00, 6.00)	221.98 (181.30, 261.10)		

Correlation between serum OSM expression and various indexes in the AIS group

**Figure 3:**
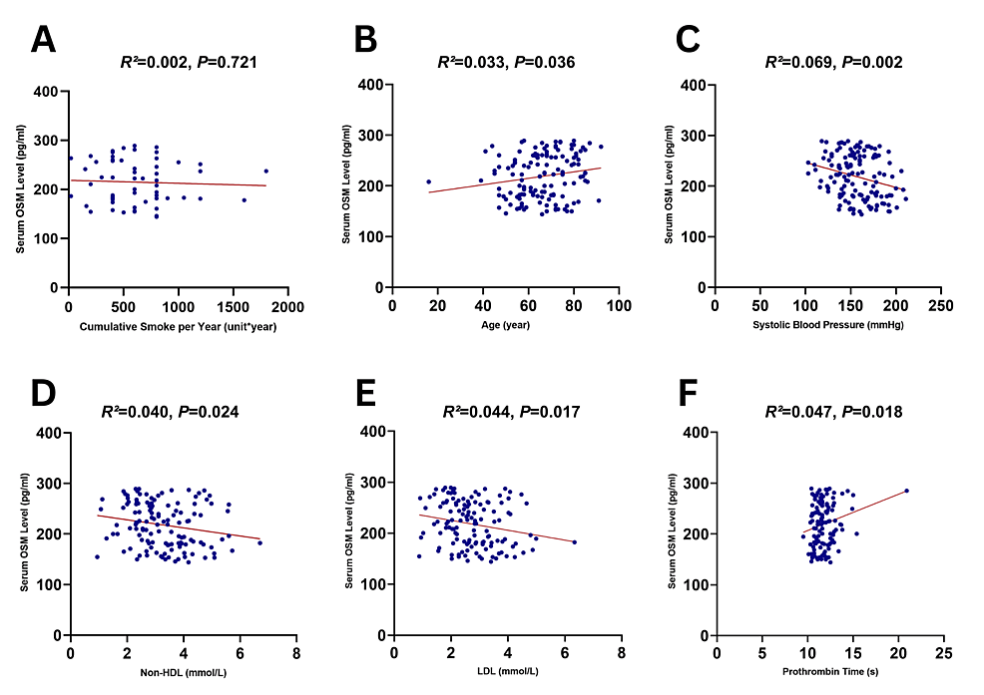
Correlation between serum OSM expression and various indexes in the AIS group non-HDL: non-high-density lipoprotein; LDL: low-density lipoprotein; AIS: acute ischemic stroke; OSM: oncostatin M A: cumulative smoking volume, B: age, C: systolic blood pressure, D: non-HDL, E: LDL, and F: prothrombin time

We compared OSM expression with various risk factors of AIS. We found that AIS patients with a history of smoking had lower serum OSM levels compared to non-smoking patients (mean 215.01 (IQR 180.83, 256.80) pg/ml vs. mean 221.81 (IQR 181.70, 260.65) pg/ml, P=0.373). We further analyzed the correlation between cumulative smoking volume and serum OSM level (in “stick * year”, if a subject consumed 20 units of cigarette per day for 20 years, the cumulative would be 20x20 stick * year), where it showed a slight negative correlation (*R*^2^=0.002, P=0.721) in the Spearman *R*^2^ correlation test, as displayed in Figure 3A. Other results showed that serum OSM correlation was significant in age, LDL, non-HDL, PT, and systolic blood pressure (Figure 3B-3F) and was not significant in gender, BMI, history of coronary heart disease, hypertension, diabetes, history of alcohol consumption, FBG, HDL, HCY, WBC, and D-D.

## Discussion

In this case-control study, we observed the serum OSM expression in AIS patients and compared it with the control group. We found that serum OSM expression was elevated in AIS patients, varying across different etiological groups, severities, and prognoses. Although there was no consistency in the pattern related to infarct volume, we found that serum OSM expression was less elevated in the group with a favorable prognosis. We also found several correlations with risk factors of AIS results were statistically significant.

A preliminary study done by Guo et al. reported a potential use of OSM for stroke treatment in the animal model [20]. Additionally, a longitudinal study by Stanne et al. reported that serum OSM was one of the most notably elevated serum expressions across the observed time frame, but to date, OSM expression had not been analyzed in clinical AIS cohort study [24]. The current study focused on the clinical implementation of OSM in AIS across two timeframes: onset and 90 days. The findings were consistent with the previous reports and provided a theoretical basis for further exploration of OSM and AIS.

The risk factors of AIS in the Chinese population have been summarized by Tu et al. [5]. We confirmed that OSM expression level was positively correlated with age, as age is one of the leading risk factors for AIS [25]. Our findings on OSM expression among different severity groups and positive correlation on PT supported the recent study by Zhao et al. [26], that OSM could also be potentially used as a novel short-term prognostic biomarker for AIS. We also found that systolic blood pressure was negatively correlated with AIS, whereas LDL and non-HDL expression shared a relatively similar negative correlation toward AIS and was statistically significant. While past studies concluded a strong dose-related relationship between smoking and AIS occurrence [25], the current study found that OSM was only slightly negatively correlated with the cumulative smoking amount.

By implementing a case-control study in a clinical setting, the present study fills the gap in OSM research related to AIS. A comprehensive set of tests was conducted to evaluate the sensitivity of the findings. To the best of our knowledge, this was the first study to investigate the association of OSM expression with the etiology, severity, prognosis, and risk factors of AIS in a clinical setting. Our study findings supported previous research on OSM and contributed to expanding our knowledge of OSM in the neurological field.

Several limitations to this study should be acknowledged. Potential confounding factors in the AIS and control groups may not have been fully matched, which potentially introduces bias into the results. Our single-time blood sample collection to measure OSM expression was unable to capture dynamic changes in OSM levels over time. OSM expression in cerebrospinal fluid was not assessed. Additionally, potential biases arising from human demographics, systematic errors, or sampling errors could potentially be mitigated by increasing the sample size.

## Conclusions

This study confirmed the elevated serum OSM expression in AIS patients. We investigated the correlation of OSM expression with the etiology, severity, and prognosis of AIS. These findings support previous research on OSM and underscore its potential utility as a novel biomarker for AIS diagnosis and treatment. Further research with a larger sample size, a longer follow-up period, and more comprehensive clinical assessments could be beneficial to gain a deeper understanding of OSM's role in AIS and pave the way for the development of OSM-targeted therapies.
